# *Lomentospora prolificans*: An Emerging Opportunistic Fungal Pathogen

**DOI:** 10.3390/microorganisms10071317

**Published:** 2022-06-29

**Authors:** Afroditi Konsoula, Constantinos Tsioutis, Ioulia Markaki, Michail Papadakis, Aris P. Agouridis, Nikolaos Spernovasilis

**Affiliations:** 1 Department of Pediatrics, General Hospital of Sitia, 72300 Sitia, Greece; aphrodite.konsoula@gmail.com; 2 School of Medicine, European University Cyprus, Nicosia 2404, Cyprus; a.angouridis@euc.ac.cy; 3 3rd Department of Internal Medicine, “Sotiria” General Hospital, 11527 Athens, Greece; tzouliamar95@gmail.com; 4 Department of Internal Medicine, “Agios Panteleimon” General Hospital of Nikaia, 18454 Piraeus, Greece; papadakis.mixal@gmail.com; 5 Department of Internal Medicine, German Oncology Center, Limassol 4108, Cyprus; 6 Department of Infectious Diseases, German Oncology Center, Limassol 4108, Cyprus; nikolaos.spernovasilis@goc.com.cy; 7 School of Medicine, University of Crete, 71303 Heraklion, Greece

**Keywords:** *Lomentospora prolificans*, opportunistic, fungal infection, immunosuppression

## Abstract

*Lomentospora prolificans* is an emerging opportunistic pathogen that primarily affects immunocompromised individuals leading to disseminated disease with high mortality rates while also causing infections in healthy populations. Successful recovery from infection is difficult due to high rates of intrinsic resistance to antifungals. Rapid and readily available diagnostic methods, aggressive surgical debridement wherever appropriate, and effective and timely antifungal treatment are the pillars for successful management. Future research will need to clarify the environmental niche of the fungus, further investigate the pathophysiology of infection and define species-specific therapeutic targets.

## 1. Introduction

*Lomentospora prolificans*, formerly known as *Scedosporium prolificans*, is a rare, highly virulent filamentous fungus that has been incriminated for numerous infections in immunocompromised as well as immunocompetent individuals [1]. *L. prolificans* is regarded as a truly emerging pathogen and several areas of uncertainty still exist. It can cause a remarkably varied range of infections and disseminated disease is almost always fatal due to its intrinsic resistance to most of the available antifungal agents [1]. The purpose of this review is to summarize the current knowledge regarding this fungus, as well as to highlight possible future directions in the study of this microorganism.

## 2. Epidemiology

*Lomentospora prolificans* presents with a wide spectrum of clinical manifestations, from superficial to disseminated infections, depending on the immune status of the infected individual. The history of the fungus begins in 1974, when Hennebert and Desai first described it with the name *Lomentospora prolificans*, as a well-recognized fungus detected in greenhouse soil [2]. The pathogenic nature of the fungus, however, was recognized ten years after its original detection, in 1984, when Malloch and Salkin found that this fungal species had the potential to cause infections in immunocompetent individuals. They isolated the pathogen from a bone biopsy specimen from a patient with osteomyelitis, and named it *Scedosporium inflatum* [3]. Later on, molecular genomic studies of DNA and RNA reassociations proved that *L. prolificans* and *S. inflatum* are the same pathogen [4], and the name *Scedosporium prolificans* became dominant [5]. The first case series of infections attributed to *L. prolificans* was reported in 1990 by Wilson et al. [6] and, subsequently, in 1991, Marin et al. reported the first disseminated infection of the pathogen [7].

The incidence of fungal infections has seen a notable increase over the last few years, affecting millions of individuals annually, with a wide range of clinical manifestations and high mortality rates. However, *L. prolificans* infection remains uncommon [8]. *L. prolificans* can infect both immunocompetent and immunocompromised patients, thus acting as both a primary and opportunistic pathogen, while it appears to come with a high degree of intrinsic resistance to many antifungal agents [8]. One review found that only 34 of 162 patients (21%) had no underlying disease; 72 of 162 (44%) had disseminated infection [1]. The same review noted an overall mortality rate of 46.9%, but the mortality rate was 87.5% in patients with disseminated disease [1].

Concerning its taxonomy, after the One Fungus = One Name movement and genomic sequencing analyses that were conducted through the study of certain genetic loci, it was concluded that the fungal genus *Scedosporium* contains 10 discrete species, which do not include *L. prolificans*, contrary to previous considerations. Therefore, the pathogen was reclassified as *L. prolificans* and the genus *Lomentospora* was restored for this species [8].

To date, the epidemiology of *L. prolificans* is still unclear [9]. The fungus has been isolated from a wide range of environmental sources such as oil-soaked soils, cattle dang, sewage, polluted waters, plants, chicken manure and other animals [8,10]. Concerning geographic distribution, the pathogen has been detected in Australia, Southern USA and European regions such as Spain, with a prominence in dry climates [8,10,11,12]. The environmental reservoirs in which the fungus thrives have not yet been recognized [12], although epidemic outbreaks have been reported [10], including cases among hospitalized immunosuppressed individuals associated with coincident hospital renovations [12]. However, it is expected that increasing awareness will help shed light on the environmental conditions that favor the fungal niche [8].

Immunocompromised patients, such as solid organ transplant and especially hematopoietic stem cell transplant (HSCT) patients, are at elevated risk for invasive *L. prolificans* infections [9]. *Scedosporium* and *Lomentospora* infections were found to account for 25% of all non-Aspergillus fungal infections in transplant recipients [13]. Other significant predictors for the development of invasive disease include acute leukemia [1], with significant mortality rates up to 77% [1,9,14], as well as neutropenia in patients with hematologic malignancies [1,9,14,15]. On the contrary, *Lomentospora* infections are rarely observed in HIV-positive patients and in patients with primary immunodeficiency [1,8]. 

Immunocompetent hosts can also be infected with *L. prolificans*, since the fungus has been observed to colonize the lungs of patients with cystic fibrosis [11], an event that usually takes place in adolescence [8]. Additionally, the pathogen has been identified as a frequent colonizer of the ear canal and the respiratory tract of patients suffering from cavitary lung disease, but without causing any symptoms [16,17].

## 3. Pathogenesis and Host Defense

The first step in the pathogenesis of *L. prolificans* is the introduction of the fungus into the host. There are two well-known routes by which the fungus enters the human body: by inhalation of airborne conidia or by traumatic inoculation of conidial cells from contaminated environmental sources [18]. Depending on the immunologic status of the host, infection may be localized, extend to the surrounding tissues (deep extension), or disseminate hematogenously to distant organs [18]. Patients with impaired bronchopulmonary anatomy, as in cystic fibrosis, bronchiectasis and lung transplantation, are susceptible to chronic airway colonization [14,19].

After infection, a crucial step in both the life cycle and the pathogenesis is the transformation of conidia into hyphae, a process called germination [20]. In healthy individuals, conidia can be cleared by the mucociliary escalator or by pulmonary alveolar macrophages [18]. If these primary defense mechanisms fail and conidia germinate and form hyphae, they can penetrate macrophages and invade cells/tissues, as well as basal membranes/extracellular matrices [20]. One study showed that the *in vitro* interaction of human lung epithelial cells with *L. prolificans* resulted in the complete destruction of the monolayer of the epithelial cells and the formation of a biofilm [21]. In addition, hyphae can infiltrate blood vessels, cause extensive tissue infarction, and lead to widespread dissemination [22].

Depending on the site of entry, the immunological response varies, and different cells are challenged to remove the conidia. Initially, the innate immune system recognizes molecular components in the fungal cell wall through pattern recognition receptors (PRRs) [23]. The PRRs induce the synthesis of pro-inflammatory cytokines, phagocytosis, and adaptive immunity mechanisms. Toll-like-receptors (TLRs), especially TLR2 and TLR4, and C-type lectin receptors, such as Dectin-1 and mannose receptors (MR), are mainly investigated as PRRs. Their importance was demonstrated by Lamaris et al.; TLR-deficient *Drosophila melanogaster* flies were susceptible to infection with *L. prolificans* and developed acute infections with high mortality rates [24]. In another study, MR and Dectin-1 receptors were found to mediate conidial uptake by central nervous system (CNS) microglial cells [25]. It should be emphasized that blocking each receptor individually successfully inhibited the process of phagocytosis, but simultaneous inhibition of the aforementioned receptors did not result in a synergistic effect [25]. Thus, it is hypothesized that other receptors may also be involved in this process [25]. 

Potential virulence factors in the fungal cell wall involved in important biological events include peptidorhamnomannan, glucosylceramide, and melanin [26]. Peptidorhamnomannan, especially the O-glycosides of the molecule, is a key determinant for fungal recognition and phagocytosis, and induces killing by macrophages and production of pro-inflammatory cytokines, such as tumor necrosis factor α (TNF-α), and nitric oxide (NO) [27]. Another bioactive molecule present on the surface of conidial and hyphal cells is glucosylceramide (GLcCer) [26]. GLcCer belongs to sphingolipids, which are essential for fungal growth, virulence, and hyphal elongation. Explicitly, purified GLcCer from *L. prolificans* activates peritoneal macrophages, leading to the production of NO and superoxide and, consequently, to conidial death [28]. Moreover, in vivo experiments have shown that purified GLcCer from *L. prolificans* was able to increase the production of pro-inflammatory cytokines by splenocytes and induce recruitment of PMNs, eosinophils, small peritoneal macrophages, and mononuclear cells to the peritoneal cavity [26,28]. However, the receptor of GLcCer has not yet been discovered. In addition, *L. prolificans* produces 1, 8-dihydroxynaphthalene melanin (DHN-melanin), which helps in evading the immune response by masking pathogen-associated molecular patterns (PAMPs), by blocking phagolysosome formation and acidification, and by interfering with host cell apoptotic pathways [26]. Targeted deletion of melanin biosynthetic genes has shown that melanin protects fungi from oxidative killing by H_2_O_2_ and UV radiation [29]. 

Recognition of the fungal molecules triggers the immune system to confront the microbial presence. Phagocytes (monocytes, neutrophils, microglia) are among the most important cells against fungal infections, as they are able to recognize and phagocytose the fungi and act as antigen-presenting cells, bridging innate and adaptive immune responses. In a study conducted by Gil-Lamaignere et al., the innate immune response was compared to the well-studied fungus, *Aspergillus fumigatus* [30]. Specifically, monocyte-derived macrophages could phagocytose *L. prolificans*, in a sense proportional to *A. fumigatus*, despite the larger size of its conidia. In contrast, the germination process of *L. prolificans* conidia was inhibited less efficiently than that of *A. fumigatus* [30]. Thus, despite the fact that conidia can be phagocytosed, they can germinate inside macrophages and form germ tube-like projections that can lyse the membrane to reach the extracellular medium. 

Neutrophils are an important component of the innate immunity regarding the control of the hyphae. Neutrophils damage hyphae mainly by degranulation by the release of large amounts of reactive oxygen species (ROS) and by the formation of neutrophil extracellular traps (NETs) that enclose microbes in a matrix of DNA and enzymes with antimicrobial activity [8,30,31]. The aforementioned susceptibility of this fungus to the innate immune system may explain its high incidence in neutropenic patients [32]. 

It has been demonstrated that phagocytes in the CNS respond poorly to this fungus and allow germination and branching of the hyphae [25]. Specifically, phagocytosis is impaired in microglial cells compared with other phagocytes, with lower release of pro-inflammatory cytokines, such as TNF and interleukin-6 (IL-6), and production of ROS [25]. Even in extremely acidic environments, as may be found inside microglial phagolysosomes, *L. prolificans* cells manage to survive pH stress and maintain high viability levels under both basic and acidic conditions [25]. Given the data above, a weak microglial response against *L. prolificans* could partially explain the propensity of this fungus to invade and live in the CNS, a phenomenon known as neurotropism [25].

A number of studies have aimed to evaluate the immunomodulatory and therapeutic effect of cytokines against *L. prolificans* [25,33]. *L. prolificans* has been shown to elicit higher synthesis of TNF-a and IL-6 by human monocytes in vitro compared with *Aspergillus* species [33]. This effect may be associated with the differences in the composition of their cell walls [33]. Similarly, Pellon et al. measured the release of these cytokines by peritoneal macrophage-like cells and microglial cells and demonstrated that macrophages produce them faster and at higher concentrations [25]. Therefore, as mentioned above, microglial response to this fungus is impaired. 

Since cytokines are produced by immune cells in response to the presence of fungi, they have been studied as therapeutic agents alone or in combination with other drugs. The granulocyte-colony stimulating factor (G-CSF) has been shown to be effective against *L. prolificans* invasion in neutropenic hosts when combined with antifungal agents [34,35]. G-CSF stimulates proliferation and differentiation of myeloid progenitor cells, resulting in increased numbers of circulating neutrophils and enhanced phagocytic response [36]. 

Other cytokines that have been studied for therapeutic purposes include granulocyte-macrophage colony stimulating factor (GM-CSF) and interferon gamma (INF-γ). GM-CSF stimulates myeloid hematopoiesis in the early stages of differentiation of myeloid cells to produce more neutrophils, eosinophils and monocytes [36], enhancing their antifungal response and the expression of TLR2 and Dectin-1 [37,38,39]. INF-γ is a crucial cytokine for the innate and adaptive immune response to invasive fungal infections, mainly because it is associated with the migration, adherence and antifungal activity of neutrophils and/or macrophages [36,40]. It has been demonstrated that INF-γ and GM-CSF in combination accelerate the antifungal activity of neutrophils by increasing superoxide production [40]. Likewise, treatment with interleukin-15 has been shown to enhance hyphal damage, release of interleukin-8 and oxidative burst of neutrophils in response to *L. prolificans* [41]. Therefore, considering the previously mentioned evidence and the susceptibility of this fungus to phagocytosis, the low incidence in immunocompetent individuals can be explained [8].

**Figure 1:**
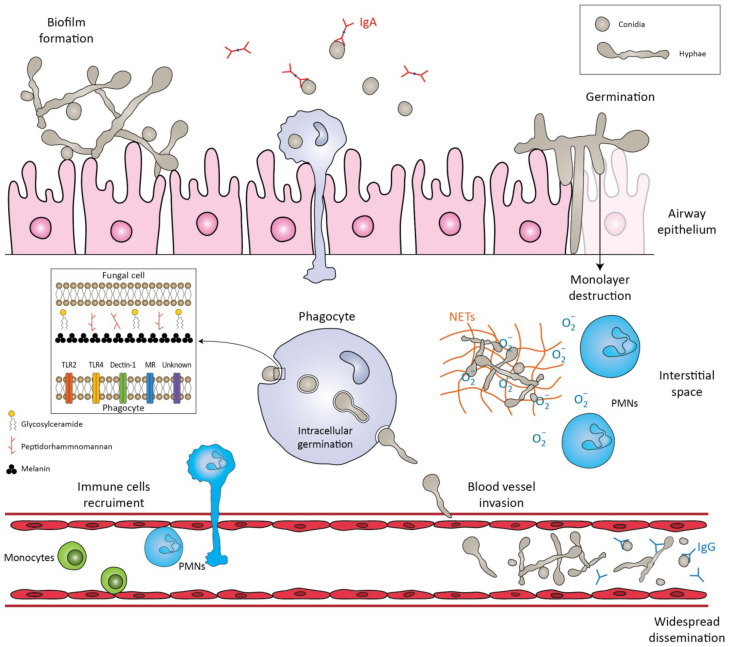
Schematic representation of the immune response against *Lomentospora prolificans*. The conidia are inhaled by the host. The mucociliary escalator and pulmonary alveolar macrophages can clear the conidia. If these primary mechanisms fail, the conidia transform into hyphae, a process called germination. The hyphae can form a biofilm, invade cell tissues/extracellular matrices, or destroy the monolayer of epithelial cells. Potential virulence factors in the fungal cell wall are peptidorhamnomannan, glycosylceramide, and melanin. Pattern recognition receptors (PRRs) that are involved in the recognition of fungus by phagocytes are TLR2, TLR4, Dectin-1, ΜR and other unknown receptors. Recognition of fungal molecules leads to the activation of immune cells in response to the microbial presence. Polymorphonuclears (PMNs) damage hyphae by degranulating reactive oxygen species (ROS) and by forming neutrophil extracellular traps (NETs). Even if phagocytosed, conidia can germinate inside phagocytes and penetrate their cell membrane. The hyphae can invade blood vessels and sporulate, leading to widespread dissemination. Salivary IgA exclusively recognizes the conidia, whereas serum IgG recognizes both forms of the fungus, conidia and hyphae.

Some of the antigenic epitopes of *L. prolificans* have recently been identified and some of the antibodies that recognize them may provide protection against this fungus [23,42,43]. Of note, human saliva containing IgA almost exclusively recognizes *L. prolificans* conidia [43], whereas serum IgG recognizes both forms of the fungus, hyphae and conidia [42]. This finding is consistent with the hypothesis of fungal invasion of the respiratory tract, in which conidia and not hyphae are inhaled by the host [43]. Saliva and serum from immunocompetent individuals were used in these studies [42,43]. Some of these antibodies produced by healthy populations may provide protection against fungal infections, and their antigenic targets may be investigated as therapeutic agents in the future [23] (Figure 1).

## 4. Clinical Presentation

Disseminated infection is by far the most frequently encountered pattern of *L. prolificans* infection and it carries a high mortality rate, as highlighted by various reviews [1,32,44,45,46]. Solid organ transplant and HSCT, malignancy, acquired immunodeficiency syndrome, neutropenia and immunosuppressive therapy, as mentioned above, are well-recognized risk factors [1,32,44,45,46]. Clinical presentation is determined by the primary focus of *L. prolificans,* by the degree of immunosuppression and by how rapidly the disease progresses. Fever, signs and symptoms of CNS and lung involvement, along with skin lesions, specifically numerous erythematous nonpruritic skin nodules with or without a necrotic center, are considered indicative of disseminated infection [18,47] and represent the most frequently encountered manifestations [1,8,46].

Respiratory tract infection by *L. prolificans* shares the same risk factors as disseminated disease. Distinguishing between colonization and infection can be difficult [1]. Colonization is defined as the presence of fungus or fungal elements in respiratory secretions without symptoms and in the absence of radiological or endobronchial changes [48]. Colonization has been studied in detail in lung transplant recipients and patients with cystic fibrosis. Structural changes in the airways, long-term immunosuppression, and previous exposure to antifungal agents contribute to the higher prevalence of *L. prolificans* in this group of patients [48,49,50]. It is worth noting that *L. prolificans* colonization constituted a contraindication to lung transplantation in several centers [51], while in a more recent study, colonization was not associated with worse survival rates [48]. Symptoms of respiratory infection include cough, dyspnea, fever and pleuritic chest pain [52].

*L. prolificans* endocarditis, while rare, carries high mortality. It is primarily observed in immunocompromised patients or patients with risk factors for endocarditis [53,54]. The mitral and aortic valve are primarily affected, although reports involving the tricuspid valve exist in the literature, one of which was associated with an implanted pacemaker [54,55]. Fever and embolic phenomena are common [53,56]. Despite the fact that blood cultures are positive in the majority of cases [54], appropriate treatment is often delayed as results are usually not readily available. Several patients underwent surgical interventions, either for valve replacement or for the removal of the infected pacemaker [53,54,57,58], often with discouraging outcomes.

CNS involvement has frequently been described in the context of *L. prolificans* infection. CNS lomentosporiosis primarily manifests as meningitis [59], meningoencephalitis [1,46,60] and brain abscess formation [61,62,63], and it is usually the aftereffect of disseminated disease. In a review of 162 cases, CNS involvement was documented in 40.3% of patients with disseminated infection, while only two patients had solely meningoencephalitis [1]. Two cases of meningoencephalitis following intrathecal drug injection [46,60] raised the suspicion that the fungus can be introduced during therapeutic lumbar puncture, highlighting the importance of aseptic conditions, especially in immunocompromised patients. Presenting symptoms include headache, nausea, signs of meningeal irritation, seizures and focal neurologic deficits [46,60,64].

Skin, soft tissue, muscle, bone and joint infections can also be caused by *L. prolificans*. In fact, in a recent systematic review, *L. prolificans* was found to be the second most common fungus to be implicated in non-*Aspergillus* osteoarticular mycoses [65]. Notably, these sites of involvement are more common in immunocompetent hosts [1] and infection usually requires disruption of the anatomic barrier by trauma [66,67,68,69,70], surgery [71,72] or corticosteroid injections [73]. Patients frequently present with pain, erythema, decreased range of motion, tenderness and edema of the affected joint while constitutional symptoms are seldom present. Rare reports of vertebral osteomyelitis with or without epidural abscess formation have been described in the literature [68,71,74], and are typically characterized by chronic and gradually progressive symptomatology. It is worth mentioning that apart from aggressive and meticulous surgical debridement and the combination of antifungal agents, antifungal agent-loaded bone cement has also shown promising results in bone infections caused by this fungus [70,73,75].

Ocular manifestations, mainly consisting of endophthalmitis, keratoscleritis and keratouveitis, are part of the disease spectrum caused by *L. prolificans*. Endophthalmitis can be both exogenous, as a result of surgical manipulation, traumatic implantation, or superficial infection, and endogenous, following hematogenous spread [76]. Endogenous endophthalmitis can occur in multiple settings, such as intravenous drug use [1,77], and disseminated disease [77,78,79,80,81]. Exogenous endophthalmitis has been reported in penetrating corneal injury [1,82]. Common presenting symptoms are decreased visual acuity, visual disturbances, photophobia and eye pain. Keratoscleritis is associated with pterygium surgery with adjunctive beta-irradiation [1,83,84], and keratouveitis is described in patients with a retained contact lens [1,85]. Foreign body sensation, conjunctival injection, lacrimation and discharge were part of the clinical presentation.

Finally, several other clinical manifestations have been linked to *L. prolificans* infection, such as mycotic aneurysms [86,87], external otitis [1,88], sinusitis [1,77,89], peritonitis [77,90], onychomycosis and esophagitis [77].

## 5. Diagnosis

Despite advances in molecular diagnostic methods, identification of *L. prolificans* from clinical specimens principally relies on direct microscopic examination of fresh specimens or histopathologic analysis, along with culture on appropriate culture media [91]. The 2019 updated definitions of invasive fungal disease (IFD) of the European Organization for Research and Treatment of Cancer/Mycoses Study Group Education and Research Consortium (EORTC/MSGERC) emphasizes that histopathologic, cytopathologic, or direct microscopic examination of fungal hyphae and/or a positive culture from an affected site are essential criteria for proven invasive fungal infection [92]. 

Histopathologic examination of an infected tissue provides strong evidence of invasive fungal infection. However, it is not possible to identify the causative pathogen without culture because different molds share the same characteristics [91]. Of note, hyphae of *L. prolificans* appear septate and are typically found in areas of inflammation, granuloma, or necrosis [93]. Infected tissue exhibits hyphae with irregular branching patterns, sometimes with branches bridging two parallel hyphae to form an H-shaped pattern [8,93]. In addition, *L. prolificans* occasionally exhibits melanized hyphae [93,94,95]. Another distinctive feature is adventitious sporulation, which is characterized by penetration of the blood vessel wall and hematogenous sporulation, resulting in positive blood cultures [61,96]. Thrombosis of the blood vessels is also common [91,93]. These features are strongly suggestive of *L. prolificans* infection but are not pathognomonic. 

Direct microscopy and culture are ineffective in early diagnosis [97], but they remain the cornerstone of proven fungal infection [92]. They both should be interpreted in the context of compatible disease and in the appropriate epidemiological setting. In addition, culture is important for in vitro drug susceptibility testing, as this fungus can be resistant to multiple antifungal agents [97]. Any clinical specimen from biopsies to sterile bodily fluids can be cultured [18,95]. A positive culture from the respiratory tract may indicate colonization and should be interpreted carefully in the proper clinical context [23]. Blood cultures are helpful in detecting disseminated infection. Notably, in a recent review, blood cultures were positive in 52 of 72 patients (72%) with disseminated infection by *L. prolificans* [1]. However, due to slow growth, most blood cultures became positive shortly before the patient’s death, thus limiting their diagnostic usefulness [1].

Molecular techniques are becoming increasingly available but should only be used as an adjunct to conventional laboratory testing [15]. Polymerase chain reaction (PCR) technology, either panfungal or species-specific, followed by DNA sequencing can identify invasive fungal infections directly from fresh and formalin-fixed paraffin-embedded (FFPE) tissue, as well as from other clinical specimens such as blood, bronchoalveolar lavage fluid, cerebrospinal fluid, and sputum [98,99,100]. Several genomic groups have been used to identify this fungus, but internal transcribed spacer (ITS) sequence seems to be the most useful [101,102]. As molecular techniques have evolved over the years, the EORTC/MSGERC has expanded the definition of proven invasive mold infection to allow identification by PCR in combination with DNA sequencing, but only after the fungus has been detected by histopathologic examination [92,103]. 

Matrix-assisted laser desorption/ionization time-of-flight (MALDI-TOF) mass spectrometry is increasingly used as a rapid and accurate identification method for *Scedosporium/L. prolificans* species [104,105]. This method uses a laser to ionize fungal proteins, which are then separated and travel through a tube according to their mass-to-charge ratio. The time it takes for the ions to travel to the detector at the end of the tube is measured. The results are compared to a database of known organisms [104]. In a recent study, MALDI-TOF mass spectrometry was able to recognize 64 *Pseudallescheria* and *Scedosporium* isolates (including *L. prolificans*) with 100% accuracy [106]. Rapid species detection allows optimization of early empirical antifungal treatment [106]. This technology is used by only a few laboratories due to limited database availability.

Serologic tests for the detection of invasive infections by *L. prolificans* are currently under investigation. The best studied panfungal biomarker is 1, 3-beta-D-glucan (BDG), a polysaccharide composed of glucose monomers linked by 1–3 glycosidic bonds, which is present in the cell wall of many fungi [107]. This biomarker can be detected in the blood of patients with invasive fungal diseases, with the exception of *Mucorales* and *Cryptococcus* infections [107]. A meta-analysis demonstrated good specificity and sensitivity of the BDG test for invasive fungal infections [108]. Therefore, detection of BDG in the serum of high-risk patients is useful when invasive fungal infection or fungemia by *L. prolificans* is suspected [109]. However, its diagnostic accuracy in *L. prolificans* has not been established yet, and results should always be interpreted in conjunction with the other diagnostic methods mentioned above [91]. Thornton et al. have developed a specific monoclonal antibody to distinguish *L. prolificans* from other filamentous fungi in histopathological specimens [110]. It targets the enzyme tetrahydroxynaphtalene reductase, which plays a role in the biosynthesis of melanin in *L. prolificans* [110]. In a recent work, Martin-Souto et al. used enzyme-linked immunosorbent assay (ELISA) to detect a specific IgG in the serum of patients with cystic fibrosis. Using a whole protein extract of *S. boydii* as the antigen, *Scedosporium* and *Lomentospora* fungal species were serologically detected in patients’ sera with high sensitivity and specificity [111]. Further studies are needed to investigate the role of novel antigen/antibody assays in serodiagnosis.

## 6. Antifungal Therapeutic Strategies

Treatment of deep infections caused by *L. prolificans* remains a rather challenging aspect, as the pathogen carries intrinsic resistance to most of the antifungal regimens used in clinical practice. The lack of new and effective antifungal agents makes the treatment of such infections even harder.

In further detail, *L. prolificans* has been described in the current literature as a pan-antifungal resistant species [11,15], with innate resistance to commonly used antifungals [112]. Voriconazole has been proposed as the initial regimen for *L. prolificans* disseminated infections, complementary to surgical removal of the infected tissue when deemed feasible [15,113]. Such suggestions come from studies that demonstrate that voriconazole has the most robust antifungal effect when compared to other regimens [16], but without offering significant reductions in mortality rates [8,15].

A potential solution is the combination of antifungal agents. In vitro studies have demonstrated that dual therapy with voriconazole and amphotericin B or echinocandins could have a synergistic effect against *L. prolificans* [112,114], while the same principles apply to combinations of various azoles (voriconazole, itraconazole, miconazole) and terbinafine, which have demonstrated well-documented synergy with beneficial outcomes [16]. The triple combination of voriconazole, amphotericin B and anidulafungin has also been tested in vitro, with reported high synergistic effects [115].

**Figure 2:**
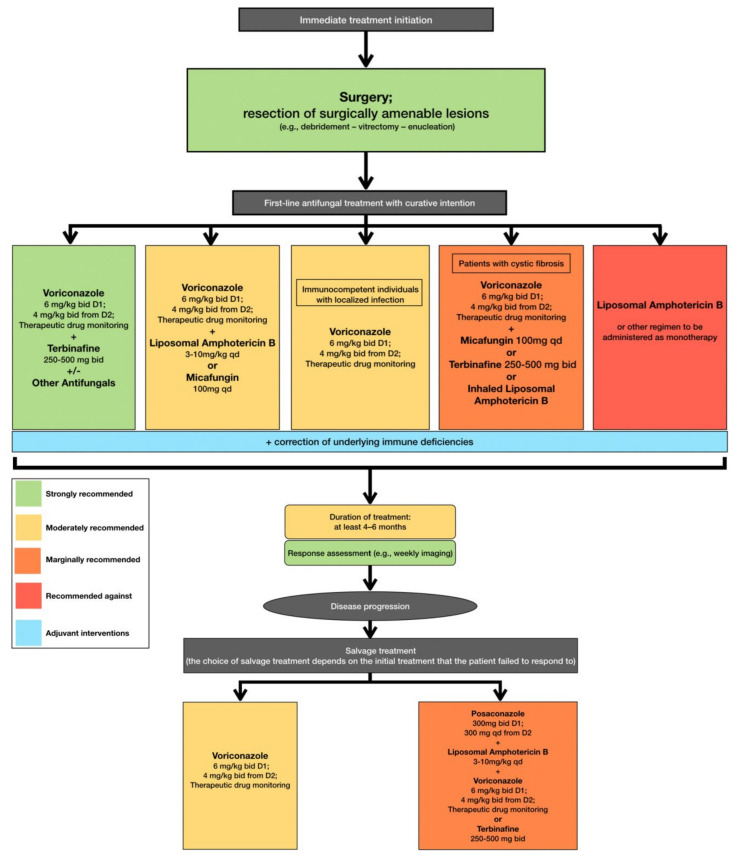
Optimal therapeutical pathway for *L. prolificans* infections in adults.

In accordance with the aforementioned, current clinical practice guidelines recommend that treatment, including surgical resection when deemed feasible, should be initiated immediately when *L. prolificans* invasive infection is confirmed or suspected [95]. Additionally, the first line of antifungal treatment should include combination therapy with voriconazole and terbinafine, whereas other combinations are only moderately or marginally recommended because there are limited data to support such a therapeutic approach [95]. Monotherapy with voriconazole should only be considered as first line treatment in immunocompetent patients with localized infection [95]. The duration of treatment is controversial, with current recommendations suggesting that combination antifungal therapy lasting at least 4 to 6 months is most likely to be associated with favorable outcomes, while it is highly recommended that response to treatment should be frequently assessed [95]. In case of disease progression, salvage treatment should be individualized and tailored to previous regimen administration [95] (Figure 2).

Schemuth et al. described the potential role of antibiotics in treating such infections, as colistin was demonstrated to have an antifungal effect when tested in vitro, either as monotherapy or when combined with antifungal regimens [116]. Moreover, Homa et al. studied the use of five antipsychotic regimens (chlorpromazine hydrochloride, trifluoperazine hydrochloride, amantadine hydrochloride, R-(-)-deprenyl hydrochloride, and valproic acid sodium salt) with antifungal activity, and their prospects in fungal infections in vitro [117]. In further detail, phenothiazines could exhibit possible antifungal properties in the treatment of locally invasive *L. prolificans* infections, while of interest remain possible combinations of antipsychotic and antifungal regimens [117].

Immune modulation interventions also play a key role in treating *L. prolificans* infections, as available data support that resolution of neutropenia in immunocompromised individuals is associated with a favorable outcome. In fact, in disseminated infection by *L. prolificans* in an immunocompromised murine model, treatment with G-CSF and liposomal amphotericin B (LAMB) improved survival compared with LAMB alone, but the improvement was not statistically significant [35]. Such data seem to also be supported by clinical experience, as there are case studies where reversion of neutropenia was linked to favorable patient outcome [34,118].

Another approach that needs further investigation is the use of adjunctive hyperbaric oxygen therapy. In a study conducted by Farina et al., in vitro tests showed that all antifungal agents had lower minimum inhibitory concentrations (MICs) when incubated with *L. prolificans* isolates in a hyperbaric hyperoxide atmosphere (100% O_2_) [119]. However, when incubated in a normal atmosphere, growth was systematically observed and MICs returned to the expected high levels [119]. Future in vivo studies may provide more information on whether hyperbaric oxygen therapy can potentially increase the antifungal activity of a single antifungal agent in order to replace the use of combination antifungal treatment.

New prospects in the treatment of disseminated resistant fungal infections are focused on the development of new pharmaceutical compounds and on deciphering molecular mechanisms through which *L. prolificans* responds to antifungal treatment. Miyazaki et al. reported that the compound E1210, a molecule that inhibits the inositol acylation step in glycosylphosphatidylinositol biosynthesis, resulting in defects in fungal cells [120], offered the potential for broader antifungal activity when compared to conventional antifungal medication, presenting with potent activity against *L. prolificans* when tested in vitro, even against azole and amphotericin B resistant strains [121]. Olorofim, the first member of the orotomide class of antifungals to be clinically tested for the treatment of such infections, has shown promising results. This molecule has the capability to inhibit dihydroorotate dehydrogenase, a key enzyme in the biosynthesis of pyrimidines. The efficacy of olorofim has been demonstrated in in vitro studies, and improved clinical outcomes were observed in two case reports [122,123,124]. The clinical efficacy of this regimen is still being tested, as the medication is currently in Phase IIB clinical trials, with existing published data coming from case reports [123,124].

Additionally, interesting data emerge from studies of the fungal molecular and structural alterations in response to antifungal treatment, as such results could shed light on yet unknown molecular cascades and potential pharmaceutical targets. In this setting, Pellon et al. studied the alterations that occur to *L. prolificans* after the administration of voriconazole, concluding that the overexpression of certain protein molecules such as heat shock proteins (HSP) could play an important role in the orchestration of antifungal drug resistance, proposing potential molecular targets for novel, more effective, antifungal compounds [125].

## 7. Conclusions

As can be deduced from available evidence so far, the most pressing issues regarding *L. prolificans* infections are the unfamiliarity of healthcare professionals with its clinical manifestations and epidemiology, the lack of or difficult to access rapid species-specific diagnostic methods, and its intrinsic resistance to most available antifungal treatments. *L. prolificans* is now considered a truly emerging, life-threatening pathogen, particularly in immunocompromised patients, raising the need for further research to address its pathophysiological, clinical and therapeutic spectrum.
